# Promoter knock-in: a novel rational method for the fine tuning of genes

**DOI:** 10.1186/1472-6750-10-26

**Published:** 2010-03-24

**Authors:** Marjan De Mey, Jo Maertens, Sarah Boogmans, Wim K Soetaert, Erick J Vandamme, Raymond Cunin, Maria R Foulquié-Moreno

**Affiliations:** 1 Ghent University, Centre of Expertise - Industrial Biotechnology and Biocatalysis, Department of Biochemical and Microbial Technology, Faculty of Bioscience Engineering, Coupure links 653, B-9000 Ghent, Belgium; 2 Ghent University, BIOMATH, Department of Applied Mathematics, Biometrics and Process Control, Faculty of Bioscience Engineering, Coupure links 653, B-9000 Ghent, Belgium; 3 Vrije Universiteit Brussel, Laboratory for Genetics and Microbiology, Department of Applied Biological Sciences, Faculty of Sciences and Bioengineering Sciences, Pleinlaan 2, B-1050 Brussels, Belgium; 4 Current address: Laboratory of Molecular Cell Biology, Katholieke Universiteit Leuven, Belgium; 5 Current address: Department of Molecular Microbiology, Flanders Institute for Biotechnology, Kasteelpark Arenberg 31, B-3001 Leuven-Heverlee, Belgium

## Abstract

**Background:**

Metabolic engineering aims at channeling the metabolic fluxes towards a desired compound. An important strategy to achieve this is the modification of the expression level of specific genes. Several methods for the modification or the replacement of promoters have been proposed, but most of them involve time-consuming screening steps. We describe here a novel optimized method for the insertion of constitutive promoters (referred to as "promoter knock-in") whose strength can be compared with the native promoter by applying a promoter strength predictive (PSP) model.

**Results:**

Our method was successfully applied to fine tune the *ppc *gene of *Escherichia coli*. While developing the promoter knock-in methodology, we showed the importance of conserving the natural leader region containing the ribosome binding site (RBS) of the gene of interest and of eliminating upstream regulatory elements (transcription factor binding sites). The gene expression was down regulated instead of up regulated when the natural RBS was not conserved and when the upstream regulatory elements were eliminated. Next, three different promoter knock-ins were created for the *ppc *gene selecting three different artificial promoters. The measured constitutive expression of the *ppc *gene in these knock-ins reflected the relative strength of the different promoters as predicted by the PSP model. The applicability of our PSP model and promoter knock-in methodology was further demonstrated by showing that the constitutivity and the relative levels of expression were independent of the genetic background (comparing wild-type and mutant *E. coli *strains). No differences were observed during scaling up from shake flask to bioreactor-scale, confirming that the obtained expression was independent of environmental conditions.

**Conclusion:**

We are proposing a novel methodology for obtaining appropriate levels of expression of genes of interest, based on the prediction of the relative strength of selected synthetic promoters combined with an optimized promoter knock-in strategy. The obtained expression levels are independent of the genetic background and scale conditions. The method constitutes therefore a valuable addition to the genetic toolbox for the metabolic engineering of *E. coli*.

## Background

The ability to alter metabolic fluxes by modifying the expression of the cognate genes is an important tool for metabolic engineering. Metabolic engineering by gene manipulation traditionally aimed at abolishing undesired metabolic activities, introducing new enzymatic activities, and/or generating many-fold overexpression of what was believed to be a rate determining step in a pathway. In some cases, these methods have been successful in redirecting the flux to a certain product but more often, the outcome has been disappointing because of the limited impact of the manipulations on the targeted result. This clearly illustrates the importance of obtaining a quantitative understanding of the factors that determine the flux through a pathway, e.g. by applying metabolic control analysis to define the best strategy for flux optimization. A comparatively straightforward approach to modify the flux through a pathway consists in the modulation of cellular enzymatic activities by changing the expression level of the corresponding genes. Several promoter libraries have been constructed for this purpose [1-11].

Bacterial genes are differentially expressed during the cell cycle in response to a wide variety of signals that modulate promoter activity and in some cases it may be interesting, however, to express specific genes constitutively and at a specific level. To ensure the constant (over)expression of a certain gene, the endogenous promoter can be replaced by a constitutive promoter with a desired strength. In this context, a synthetic promoter library is useful for the fine tuning of genes. To date, there are comparatively few data about the insertion procedure of artificial promoters directly in the chromosome [1,8]. The existing methods contain elements of randomness, in the sense that they create a collection of promoters for each gene of interest that needs to be screened for the appropriate expression. In a next step, the chromosomal promoter can be replaced by an artificial one specifically constructed for the targeted gene. Therefore, a more direct, less time-consuming approach to modify promoter strength is desirable. The present method optimizes the insertion procedure of promoters selected from a previously characterized library. We describe an optimized procedure for the fine tuning of gene expression, using as proof of concept the *ppc *gene of *Escherichia coli *(coding for phosphoenolpyruvate carboxylase). The advantage of this procedure over the state-of-the art procedures is the universal applicability of the created artificial promoter library in combination with a promoter strength predictive (PSP) model [3], making it unnecessary to create a new promoter library for each targeted gene. Hence, the proposed strategy is less time consuming and cheaper.

First, we compare several knock-in strategies of upstream untranslated regions containing a synthetic promoter with or without a synthetic canonic ribosome binding site (RBS) and with or without an N-terminal polyhistidine sequence (His-tag). Second, we apply the optimized procedure to knock-in synthetic promoters with different strength, selected using a previously developed promoter strength predictive (PSP) model. At the same time, this work constitutes a validation of the PSP model [3]. Third, we investigate the possible influence of the genetic background and scale up conditions on the expression. This is important to exclude the possibility that the expression of our proof of concept, *ppc*, is controlled by a regulator which can bind on an unknown transcription factor binding site located within the used artificial promoters or the flanking regions in the genome.

## Results

### Selection of synthetic promoters for the optimization of the knock-in procedure, using the PSP model

The PSP model was developed to quantitatively predict the strength of promoters on the basis of the nucleotide sequence [3]. A relative strength of 0.20 was calculated with this model for the endogenous promoter of the *ppc *gene (*ppc*p) in a scale from 0 to 1.00 defined by a library of 42 synthetic promoters. Out of the library, two stronger synthetic promoters, p37 (0.82) and p55 (0.36), were chosen for the knock-in experiments.

### Knock-in strategies to insert promoters

**Figure 1:**
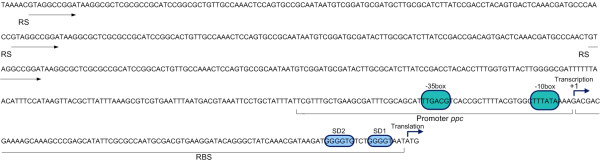
**The natural *ppc *promoter**. RBS: Ribosome Binding Site; RS: Repetitive sequences indicated by arrows; putative transcription factor binding site is underlined with a discontinuous line.

The *ppc*p was chosen to be rationally replaced. Upstream of the *ppc *gene, there is a 91 nt leader region harboring two RBS sequences, a promoter region with -10 and -35 boxes [12], and three repeated sequences (from -62 to -506) (figure 1). The relevance to *ppc *expression of the repeated sequences is not known.

**Figure 2:**
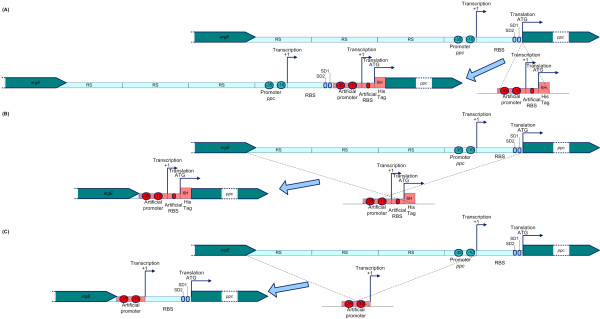
**Strategies to replace the natural *ppc *promoter by an artificial promoter**. (a) Strategy I: insertion of a promoter + artificial RBS and a polyHis-tag sequence between the *ppc *gene and its natural promoter (b) Strategy II: replacement of natural promoter by a promoter + artificial RBS and a polyHis-tag sequence (c) Strategy III: replacement of natural promoter by artificial promoter, keeping the natural RBS.

In a first attempt, a cassette containing the antibiotic resistance marker, the synthetic p37 promoter, a canonical RBS, a start codon and a polyhistidine sequence (tag) substituted the start codon of the *ppc *gene (figure 2a). The obtained mutant was grown in triplicate at flask scale to compare gene expression at transcriptomic and enzymatic level to the wild-type (wt), (table 1). The insertion of the artificial promoter resulted in a 2-fold reduction of expression of the *ppc *gene. Therefore, a second strategy was designed to replace the sequence from position -506 nt to the start codon (+94) by the p37 promoter with an artificial RBS and a start codon followed by a polyhistidine sequence (figure 2b) to eliminate the influence of a putative transcription factor binding site [13] which is located upstream of the endogenous promoter on the artificial promoter. Interestingly, an even stronger reduction in the mRNA level (5- to 6-fold) compared to the wt was observed (table 1). To exclude that the observed reduction of expression was due to the presence of the polyhistidine sequence, the latter approach was repeated by inserting the p37 promoter with its RBS but without the polyhistidine sequence (not shown in the diagram). However, a similar reduction of expression was observed (0.3 (0.2-0.4)). A possible explanation is that the synthetic leader region was not adequate to ensure mRNA stability [14]. Consequently, a third strategy was designed, respecting the natural RBS region, with introducing neither the polyhistidine sequence nor the synthetic RBS. The cassette with the p37 promoter replaced the sequence from -506 to -1 (figure 2c). This approach resulted in a successful 3- to 4-fold increase in expression of the *ppc *gene (3.9 compared to 1.0). A similar increase was observed at the enzymatic level (3.33 versus 1.00) (table 1). These results are in agreement with what was predicted by the PSP model.

**Table 1:** Expression of the *ppc *gene with the artificial p37 promoter inserted with the 3 different strategies at transcriptomic level (qPCR) and enzyme expression level.

	Strategy I		Strategy II		Strategy III	
	mRNA (2^-ΔΔct^)	PEP carboxylase activity	mRNA (2^-ΔΔct^)	PEP carboxylase activity	mRNA (2^-ΔΔct^)	PEP carboxylase activity
**Wild-type**	1.0 (0.8-1.3)	1.00 ± 0.002	1.0 (0.7-1.5)	1.00 ± 0.002	1.0 (0.9-1.2)	1.00 ± 0.002
**mutant**	0.4 (0.3-0.5)	0.50 ± 0.003	0.2 (0.1-0.3)	0.13 ± 0.001	3.9 (3.3-4.5)	3.32 ± 0.015

### Verification of the promoter knock-in procedure and of the PSP model

**Figure 3:**
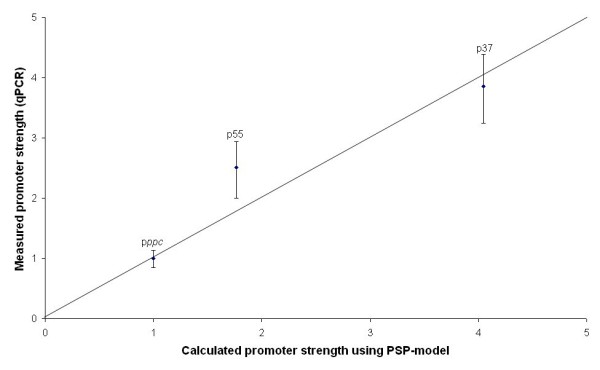
**Relation between the relative promoter strength of the natural *ppc *promoter and two artificial promoters (p55 and p37) predicted using the PSP-model and measured using qPCR**.

Two synthetic promoters (p37 and p55) were chosen on the basis of their relative strength to replace the natural *ppc *promoter. After optimizing the promoter knock-in procedure with the p37 promoter, the same approach was followed with the p55 promoter. The expression of the *ppc *gene in the wt and the mutant was measured as before. A 2.5-fold increase of *ppc *expression was observed (table 2). As shown in figure 3, there is a good correlation between the relative expression from the synthetic promoters at both transcriptomic and enzymatic activity levels and the predicted promoter strengths using the PSP model.

**Table 2:** Expression of *ppc *gene with the artificial promoters relative to the natural promoter in strain MG1655 (wt) and MG1655 Δ*ackA-pta*, Δ*poxB *(3KO).

Strain	Flask-scale		Bioreactor-scale	
	Expression of *ppc *relative to WT		Expression of *ppc *relative to WT	
	mRNA (2^-ΔΔct^)	PEP carboxylase activity	mRNA (2^-ΔΔct^)	PEP carboxylase activity
**wt**	1.0 (0.9-1.1)	1.00 ± 0.002	1.0 (0.9-1.1)	1.00 ± 0.002
**3KO**	0.7 (0.5-0.9)	0.82 ± 0.003	0.8 (0.7-0.9)	0.92 ± 0.005
**wt - p37**	3.9 (3.3-4.5)	3.33 ± 0.015	6.7 (5.8-7.8)	5.24 ± 0.013
**wt - p55**	2.5 (2.1-3.0)	1.79 ± 0.002	5.0 (4.7-5.2)	3.22 ± 0.014
**3KO - p37**	3.7 (3.3-4.3)	3.56 ± 0.002	5.7 (5.3-6.2)	4.71 ± 0.019
**3KO - p55**	2.3 (2.0-2.7)	2.02 ± 0.016	4.2 (3.9-4.5)	2.55 ± 0.007

Next, the influence of the genetic background on the expression of *ppc *from the synthetic promoter was investigated. The p37 and p55 promoters were knocked-in in an *E. coli *MG1655 mutant in which three genes coding for enzymes involved in the pyruvate oxidation pathway were knocked-out. The expression of the *ppc *gene in the mutant strains was measured relative to the wt strain at transcriptomic and enzymatic activity level (table 2). The expression of the *ppc *gene in the 3KO strain seems to be down regulated compared to the wt strain (0.7 versus 1.0) despite the fact that no regulatory elements have yet been identified in the *ppc *operon. According to Lesnik and coworkers (2005) [13], this may be due to a putative transcription factor binding site that is located upstream of the *ppc *promoter. These results seem to indicate that the introduction of additional knock-outs (blocking the acetate pathway and the pyruvate oxidizing pathway) may create a novel phenotype of which the *ppc *expression is down regulated compared to the wt. Hence, this new phenotype probably influences a regulator which binds to this putative transcription factor binding site. Furthermore, these results confirm that the relative strength of both p37 (3.9 and 3.7) and p55 (2.5 and 2.3) promoters was maintained in both the wt and the 3KO strains, respectively. Moreover, the results at enzymatic level had the same tendency (3.3 - 3.6 for promoter p37 and 1.8 - 2.0 for promoter p55 in the wt - 3KO strains).

Next, the influence of growth parameters (oxygen supply, pH and culture volume (scaling up)) on the strength of the knocked-in synthetic promoters in both the wt and the 3KO strains was investigated using a bioreactor. Again, a similar relative expression of the *ppc *gene was observed at both transcriptomic and enzymatic activity levels, showing that scaling up the culture conditions affect the strength of the synthetic promoters keeping the same tendency (6.7 - 5.7 for promoter p37 and 5.0 - 4.2 for promoter p55 in the wt - 3KO strains) (table 2).

## Discussion

Several methods have been developed to engineer the cellular metabolism by modifying the expression level of genes coding for enzymatic steps that are considered either rate-limiting or diverting the metabolic flow towards by-products. One such approach is to modify the promoter of genes using a promoter library. The existing methods [8,15] generally yield a collection of mutants which needs to be screened to select the mutant with the desired expression level since the expression cannot be predicted in advance. Hence, in a recent review, Santos and Stephanopoulos (2008) [16] stressed the need for a rational approach for the application of existing promoter libraries. In the present work, we developed a method based on the use of a previously constructed library of promoters [3] in combination with a mathematical model (Promoter Strength Predictive, PSP) which can be applied for the fine tuning of gene expression, using the *ppc *gene (phosphoenolpyruvate carboxylase) as model system. Three methodological aspects were considered: (i) the optimization of the knock-in procedure; (ii) the capability of the PSP model to compare the strength of specific promoters in a collection with a native promoter; (iii) the stability of the engineered expression level when genetic background (additional mutations) and growth conditions (scaling up) are changed.

So far, little information is available about an efficient method for the insertion of artificial promoters at the chromosomal level. The multiple mutants obtained using existing libraries require extensive screening steps. Alper and co-workers (2005) [15] used their characterized library of promoters to modulate the expression at chromosomal level for fine tuning the expression of both phosphoenolpyruvate carboxylase and deoxy-xylulose-P. The strength of the artificial promoters was assayed with indirect methods (biomass yield and lycopene production, respectively). They concluded that the optimal gene expression levels are variable and dependent on the genetic background of the strain to achieve a specific phenotype. However, they did not collect any transcriptomic nor proteomic data. We are proposing a new method that allows to evaluate the strength of the inserted promoter compared to the endogenous promoter in advance. In addition, the obtained expression is independent of additional genetic modifications and scaling up conditions.

(i) Optimization of the knock-in procedure. In order to maintain the expression of the chosen promoter it is imperative to conserve the leader region (from transcriptional to translational starting point) of the gene of interest and to eliminate upstream regulatory elements (transcription factor binding sites) (Strategy III). Simply inserting a synthetic promoter between the coding region and the natural promoter region (strategy I in figure 2) or substituting the endogenous promoter region by the same synthetic promoter fragment (Strategy II in figure 2) results in a drastic decrease in expression (2- to 3-fold and 5- to 6-fold, respectively). An N-terminal polyhistidine coding sequence, to allow an eventual quick purification of the protein of interest, was present in the two constructs. However, omitting this sequence in another construct did not improve expression. Substituting the natural promoter region 506 nt upstream of the transcription start while retaining the *ppc *gene 5' untranslated region (leader region) resulted in a 3-fold increase of expression, at both transcriptomic and enzymatic activity levels (table 1). Thus, a level of transcription corresponding to that predicted by the PSP model was obtained and, importantly, the increase in mRNA levels was translated into an increase in protein activity. Incidentally, this indicates that there is no post-translational regulation of the *ppc *gene. It is known that the untranslated sequence at the 5' end of mRNA, beyond its role in engaging ribosomes, is important for the stability of the transcript. Changes in this sequence may therefore affect the expression of the downstream gene, which seems to have been the case here with constructions I and II. This was also observed by Solem and Jensen in their gene expression modulation experiments [17]. It should be noted that in order to knock-in the artificial promoters successfully in front of the *ppc *gene, the repetitive sequences upstream of the endogenous promoter had to be eliminated as well. Hence, the putative transcription factor binding site which was previously described by Lesnik and coworkers (2005) [13] was removed. As the purpose here is to obtain the stable constitutive expression of a cellular activity, the elimination of this (putative) regulatory site is desirable.

(ii) The expression levels obtained with the two synthetic promoters from our library (p37 and p55) confirm the use of the PSP model to choose promoters in function of the desired level of expression. Indeed, the expression levels relative to wild-type were in quite good agreement with the predicted relative strengths.

(iii) The engineered *ppc *expression was independent of the genetic background, insofar as the introduction of additional mutations affecting connected metabolic branches did not affect the expression from the two promoters. The relative strength of expression remained the same in a strain in which three genes (the *ackA, pta*, and *poxB *genes involved in the pyruvate oxidation pathway) were knocked out. Changing the growth conditions by scaling up from shake-flasks to 1.5 L bioreactor conditions did not affect expression either. Furthermore, our promoter knock-in procedure ensures constitutive expression.

In conclusion, a rational approach for the modulation of gene expression, using a library of promoters in combination with a mathematical model, is proposed. The developed promoter knock-in method ensures the stable expression of the targeted gene. In addition, the knock-in procedure is almost "seamless", leaving only an 84 nt insert with no selection markers such as antibiotic resistance genes in the genome. Therefore, the method does not impose limitations on the further introduction of other synthetic promoters for the fine tuning of several other genes expression in the same *E. coli *strain.

We also applied this method to create a selection host for the detection of L-ribose isomerase expressing mutants of *Escherichia coli *[18] and to fine tune the expression of the membrane transport protein *dcuC *[19]. This indicates that the proposed procedure is also applicable for other genes.

## Conclusion

State of the art methods for the utilization of existing promoter libraries prove to be suboptimal for the fine tuning of gene expression and therefore there is a need for a rational promoter knock-in method. In this study, we demonstrated the usefulness of an existing promoter strength predictive model (PSP) to compare in advance the relative strength of the promoters in the library with the native promoter so that the latter can be replaced with an appropriate one of the former. Further, we developed an optimal strategy to knock-in promoters. Existing methods are time consuming and expensive since they involve post-insertion screening steps. We present a novel method in which the strength of the inserted promoter relative to the natural one is known beforehand and in which the obtained expression is independent of genetic background. The method is therefore a valuable addition to the *E. coli *metabolic engineering toolbox.

## Methods

### Bacterial strains and plasmids

The wild-type (wt) strain *Escherichia coli *MG1655 [λ^-^, F^-^, *rph*-1, *rfb*-50, *ilvG*^-^, *fnr*^-^] was obtained from the Netherlands Culture Collection of Bacteria (NCCB, Utrecht, The Netherlands). The mutant strain *E. coli *MG1655 Δ*ackA-pta*, Δ*poxB *[λ^-^, F^-^, *rph*-1, *rfb*-50, *ilvG*^-^, *fnr*^-^, Δ*ackA-pta*, Δ*poxB*] was constructed using the method of Datsenko & Wanner (2000) [20] and it is referred to as 3KO. The plasmids pKD46 (Red helper plasmid, Ampicillin resistance), pKD3 (containing an FRT-flanked chloramphenicol resistance (*cat*) gene), pKD4 (containing an FRT-flanked kanamycin resistance (*kan*) gene), and pCP20 (expressing FLP recombinase activity) were obtained from Prof. Dr. J-P Hernalsteens (Vrije Universiteit Brussel, Belgium). The artificial promoter library was constructed by De Mey et al. (2007) [3]. The plasmid pBluescript (Fermentas, St. Leon-Rot, Germany) was used to create the promoter delivery constructs.

### Culture conditions

The culture medium Luria Broth (LB) consisted of 1% tryptone-peptone (Difco, Erembodegem, Belgium), 0.5% yeast extract (Difco) and 0.5% sodium chloride (VWR, Leuven, Belgium). The pH of the medium was 6.7.

For flask cultures, minimal medium (MM-flask) consisted of 18 μM FeCl_2_.4H_2_O (Merck, Leuven, Belgium), 34 μM CaCl_2_.2H_2_O (Merck), 8.3 μM MnCl_2_.2H_2_O (Merck), 2.2 μM CuCl_2_.2H_2_O (Sigma, Bornem, Belgium), 2,1 μM CoCl_2_.6H_2_O (Merck), 6.9 μM ZnCl_2 _(Merck), 0.4 μM H_3_BO_4 _(Merck), 40.3 μM Na_2_EDTA.2H_2_O (Fluka, Bornem, Belgium), 3 μM thiamine HCl (Sigma), 0.4 μM Na_2_MoO_4_.2H_2_O (Fluka), 37.4 mM NH_4_Cl (Merck), 37.8 mM (NH_4_)_2_SO_4 _(Merck), 22 mM KH_2_PO_4 _(Acros, Geel, Belgium), 42 mM K_2_HPO_4 _(Acros), 40 mM MOPS (Sigma), 2 mM MgSO_4_.7H_2_O (Fluka), 8.6 mM NaCl (VWR) and 83.3 mM glucose.H_2_O (Stop, Dendermonde, Belgium). The pH was set at 7.0 with a 1 M K_2_HPO_4 _(Acros) solution.

For batch cultures, the minimal medium (MM-batch) composition was identical to MM-flask, except for the concentration of KH_2_PO_4 _14.7 mM, and the absence of MOPS. The pH was not set to 7.0, but left at approx 5.4.

A preculture from a single colony was grown in 5 ml MM-flask medium overnight and 2 ml was transferred to 100 ml MM-flask medium in a 0.5 l flask. Incubation was performed at 37°C in a rotary shaker (160 rpm) for 16 hours. The inoculum was set at OD_600 _= 0.5, and 75 ml was used to inoculate 1.5 l MM-batch in a Biostat M fermentor (Sartorius Stedim Biotech S.A., Melsungen, Germany). In batch cultures, the pH (7.0) was kept constant using 4 N KOH and 1 N H_2_SO_4_, the temperature, agitation and air supply were set at 37°C, 1000 rpm, and 1.5 l/min, respectively. The pH, pO_2_, temperature, agitation, used acid and used base were followed online using the MFCS/WIN software of Sartorius Stedim Biotech S.A. Samples were taken using a rapid sampling loop. Each hour, a sample for OD_600 _and extracellular measurements was taken using the stainless bead sampling method as described by Mashego *et al. *[21], followed by cold centrifugation. During exponential growth, a sample was taken every 30 min. At OD_600 _= 1, 1 ml samples were taken for total RNA extraction and enzymatic activity measurements.

### Promoter delivery plasmids

Two selected promoters (p37, and p55) (table 3) were amplified from an existing promoter library [3] by PCR with primers (Fw-EcoRI-p37/Rv-BamHI-p37, and Fw-EcoRI-p55/Rv-BamHI-p55, respectively) flanked with restriction site regions (EcoRI and BamHI) (table 3). The antibiotic resistance genes (for chloramphenicol or kanamycin resistance) flanked with FRT sites were amplified by PCR with primers (Fw-EcoRI-P1/Rv-HindIII-P2) carrying the restriction site regions (EcoRI and HindIII) and priming sites from pKD3 and pKD4, respectively. The PCR products were digested with the appropriate restriction enzymes and introduced in a vector (p-Bluescript) previously linearised (BamHI and HindIII). After verification of the promoter sequence, the final plasmid was used as template in the promoter knock-in procedure.

**Table 3:** Sequences of used promoters and primers

Primer	Sequence
**Fw-EcoRI-p37**	gggggaattccttacatgaaaaaggttcttg
**Rv-BamHI-p37**	ttttggatcccatctttgtttcctccgagaaaaatgacatataccacatgg
**Fw-EcoRI-p55**	gggggaattccttagaaggaatttgttcttg
**Rv-BamHI-p55**	ttttggatcccatctttgtttcctccgagatacctaaaaattatacc
**Fw-EcoRI-P1**	ttttgaattcgtgtaggctggagctgcttc
**Rv-HindIII-P2**	ggggaagcttcatatgaatatcctccttag
**Fw-ppc-HIS-RBS-37**	acattactacgcaatgcggaatattgttcgttgtggtgatggtgatggtgcgccatctttgtttcctccgagaaaaatgac
**Fw-ppc-HIS-RBS-55**	acattactacgcaatgcggaatattgttcgttgtggtgatggtgatggtgcgccatctttgtttcctccgagatacctaa
**Rv-ppc-P2**	cgtgaaggatacagggctatcaaacgataagatggggtgtctggggtaatcatatgaatatcctccttag
**Rv-ppc-3-P2**	atcaagcccacccgcgaactgataacccaggtaattcaccatttttgctggcattaacatatgaatatcctccttag
**Fw-ppc-37**	tccttcacgtcgcattggcgcgaatatgctcgggctttgcttttcgtcgtcaaaaatgacatataccacatgga
**Fw-ppc-55**	tttgccgagcatactgacattactacgcaatgcggaatattgttcgttcatctttgtttcctccgagatacctaaaaattataccacatcaac

### Promoter knock-in procedure

The promoter knock-in system is based on the λ Red-mediated one step recombination procedure for creating a knock-out mutant as described by Datsenko and Wanner [20], with several modifications. The basic strategy is to replace a chromosomal sequence with a cassette containing a selectable antibiotic resistance gene and the sequence to be inserted. The cassette is generated by PCR by using primers with circa 50 nt homology extension (H1 and H2).

### Strategies for the insertion of an artificial promoter

The design of the primers (table 3) for the replacement of the endogenous *ppc *promoter (*ppc*p) was based on different strategies: (i) insertion of an artificial promoter, plus an artificial ribosomal binding site (RBS) and a polyHis-tag sequence between the *ppc *gene and the *ppc*p (Fw-ppc-HIS-RBS-37(55)/Rv-ppc-P2); (ii) replacement of *ppc*p by an artificial promoter, an artificial RBS and a polyHis-tag sequence (Fw-ppc-HIS-RBS-37(55)/Rv-ppc-3-P2); (iii) replacement of *ppc*p by an artificial promoter, but respecting the transcriptional starting point and the natural RBS (Fw-ppc-37(55)/Rv-ppc-3-P2). In strategies I and II, a polyHis-tag was included to facilitate detection and purification of the protein.

### Quantitative PCR

The wild-type and the mutants were grown in flasks in 20 ml MM-flask medium in triplicate. One ml samples were collected at OD_600 _= 1.0 for mRNA and protein collection. Total RNA extraction was done using the RNeasy mini kit of Qiagen^® ^(KJ Venlo, The Netherlands). The purity of RNA was verified on a FA-agarose gel as recommended by Qiagen^® ^and the RNA concentration was determined by measuring the absorbance at 260 nm. 2 μg RNA was used to synthesize cDNA using a random primer and RevertAid H Minus M-MulV reverse trancriptase (Fermentas).

The strength of the promoter was determined by RT-qPCR carried out in an iCycler IQ^® ^(Bio-Rad, Eke, Belgium) using the primers Fw-ppc-qPCR and Rv-ppc-qPCR. SYBR GreenER qPCR supermix (Invitrogen^®^) was used to perform a brief UDG (uracil DNA glycolsylase) incubation (50°C for 2 min) immediately followed by PCR amplification (95°C for 8.5 min; 40 cycles of 95°C for 15 s and 60°C for 1 min) and melting curve analysis (95°C for 1 min, 55°C for 1 min and 80 cycles of 55°C+0.5°C/cycles for 10 s) to identify the presence of primer dimers and analyze the specificity of the reaction. This UDG incubation step before PCR cycling destroys any contaminating dU-containing products from previous reactions. UDG is then inactivated by the high temperatures during normal PCR cycling, thereby allowing the amplification of genuine target sequences. Each sample was performed in triplicate. The relative expression ratios were calculated using the "Delta-delta ct method" of PE Applied Biosystems (Perkin Elmer, Forster City, CA). The gene *rpoB *was used as housekeeping gene using the primers Fw-rpoB-qPCR and Rv-rpoB-qPCR.

### Phosphoenolpyruvate carboxylase assay

Cell lysis was performed with the EasyLyse™-kit (Epicentre^® ^Biotechnologies, BIOzymTC, Landgraaf, Netherlands), following the procedure recommended by the supplier. PEP carboxylase activity was assayed as described by De Maeseneire *et al. *[22]. The absorbance at 415 nm was measured in a microplate reader (680 XR microplate reader, Bio-Rad, Eke, Belgium). PEP carboxylase activity was measured in the knock-in mutants and in the wild-type.

## List of abbreviations used

3KO: *E. coli *MG1655 Δ*ackA-pta*, Δ*poxB*; FRT: FLP recognition target; KI: knock-in; KO: knock-out; PEP: phosphoenolpyruvate; PLS: partial least square; *ppc*p: promoter of the *ppc *gene; PSP model: promoter strength predictive model; RBS: ribosome binding site; RS: Repetitive Sequence; wt: wild-type.

## Authors' contributions

JM applied the PSP model to calculate the promoter strengths. MDM, MRFM and SB carried out the molecular genetic studies. MDM and MRFM drafted the manuscript. EJV, RC and WKS revised the manuscript critically. All authors read and approved the final manuscript.
